# Extended minimally invasive autopsy: Technical improvements for the investigation of cardiopulmonary events in COVID-19

**DOI:** 10.6061/clinics/2021/e3543

**Published:** 2021-11-17

**Authors:** Jair Theodoro-Filho, Renata Aparecida de Almeida Monteiro, Amaro Nunes Duarte-Neto, Thais Mauad, Luiz Fernando Ferraz da Silva, Paulo Hilário Nascimento Saldiva, Marisa Dolhnikoff

**Affiliations:** Laboratorio de Investigacao Medica (LIM-05), Departamento de Patologia, Faculdade de Medicina FMUSP, Universidade de Sao Paulo, Sao Paulo, SP, BR.

**Keywords:** Autopsy, Minimally Invasive Autopsy, COVID-19

## Abstract

**OBJECTIVES:**

Ultrasound-guided minimally invasive autopsies (MIA-US) are an alternative to conventional autopsies and have been used in our institution to investigate the pathophysiology of COVID-19 since the beginning of the pandemic. Owing to the limitations of post-mortem biopsies for evaluating cardiopulmonary events involving large vessels, we continuously improved the technique during this period. Objectives: To demonstrate the usefulness of an extended MIA-US technique (EMIA-US) for the study of thoracic involvement in COVID-19.

**METHOD:**

US-guided percutaneous tissue sampling was combined with a small thoracic incision (≤5 cm), allowing for the sampling of larger tissue samples or even the entire organ (lungs and heart).

**RESULTS:**

EMIA-US was performed for eight patients who died of COVID-19 in 2021. We demonstrate cardiopulmonary events, mainly thromboembolism and myocardial infarction, that could be evaluated using EMIA-US.

**CONCLUSIONS:**

Minimally invasive image-guided post-mortem tissue sampling is a flexible and practical method to conduct post-mortem studies of human diseases, mainly in areas that do not have autopsy facilities or, alternatively, when autopsy is not possible owing to financial constraints, cultural and religious values, or for safety reasons, such as in the case of highly contagious infectious diseases. We present evidence that EMIA-US is feasible and can be used as an alternative to increase the accuracy of MIA-US in detecting cardiopulmonary events involving large vessels, which may not be assessed through post-mortem biopsies.

## INTRODUCTION

Minimally invasive autopsy (MIA) is an alternative to conventional autopsies in some situations. In areas devoid of autopsy facilities, MIA is used to establish the cause of death, especially in the context of infectious conditions (1,2). The portability of MIA offers a unique opportunity to conduct autopsy in distant settings such as that in Brazil since the 1920s, a period in which a special device, the viscerotome, was employed in the investigation of the pathogenesis of yellow fever and other infectious diseases, such as leishmaniasis and schistosomiasis (3).

Briefly, MIA involves obtaining tissue samples from different organs via percutaneous puncturing (1,2). It can be performed using external anatomical references or under image guidance. Imaging—magnetic resonance, computed tomography (CT), or ultrasound—increases the sensitivity of MIA (2,4). In our institution, ultrasound-guided MIA (MIA-US) has been employed since 2016 in selected cases (5). When the COVID-19 pandemic emerged, MIA-US was the only option available to conduct autopsies, since conventional autopsies were interrupted owing to the risk of contagion. From the beginning of the pandemic, our team performed 252 MIA-US procedures; 117 of these patients died of COVID-19 (confirmed by RT-PCR and/or immunohistochemistry). The remaining 135 cases comprised 71 with other clinical conditions and 64 of intrauterine or neonatal deaths. The study of COVID-19 cases provided information on different aspects of the pathophysiology of the severe form of the disease, such as the characterization of pulmonary injury (6), thrombogenic potential of COVID-19 (7), timeline of pulmonary remodeling (8), systemic distribution of the viral infection (6,9), and peculiar characteristics of severe COVID-19 in children (9).

Owing to limitations of MIA-US for evaluating specific clinical conditions, such as cardiopulmonary events involving large vessels, during the pandemic, we continuously improved the technique to refine the investigation of thromboembolic events and myocardial infarction, important presentations and complications of severe COVID-19. We have developed an alternative approach, in which conventional percutaneous tissue sampling is combined with a small thoracic incision, allowing sampling of a larger amount of tissue than that in post-mortem biopsies, or even the removal of the entire organ. In the present report, we exemplify the usefulness of this extended MIA-US (EMIA-US) technique for the study of thoracic involvement in COVID-19.

## METHODS

The images presented herein were obtained from autopsies of patients who died of COVID-19 at HC-FMUSP in 2021, in the city of Sao Paulo, Brazil. This protocol was approved by the HC-FMUSP Ethical Committee (protocol no. 3951.904). The procedures were performed in accordance with the ethical standards of the Ethical Committee on Human Experimentation and with the Helsinki Declaration.

The conventional MIA-US protocol is based on the percutaneous tissue sampling of several organs under ultrasound guidance (10). Briefly, images obtained using a portable ultrasound (SonoSite M-Turbo R, Fujifilm, Bothell, WA, USA) were used to localize and orient sampling of several organs and to select the most affected areas within each organ. Tissue sampling was performed using Tru-Cut semi-automatic coaxial needles (14G; 20 cm in length) (6,9,10). In EMIA-US, we complement the conventional MIA-US approach by performing a small (≤5 cm) thoracotomy between the left 3^rd^ and 4^th^ intercostal spaces. Subcutaneous tissue was separated from the rib cage, and two to three ribs were dissected and extracted from the costal cartilage up to 4 cm before reaching the subcostal groove. After opening the rib cage, the pericardium was opened, and the heart was exposed. Subsequently, the left mediastinal pleura was carefully sectioned to expose the left lung. The pulmonary hilum was exposed, and the main bronchus, pulmonary artery, and veins were sectioned 2 cm away from the heart, allowing the removal of the whole organ. The left lung was then gently pulled from the rib cage to be removed entirely. Using the same left incision and dissection procedures, the right lung was removed as well. The thorax was then filled with sawdust as in conventional autopsies, and a subcutaneous dermal suture was performed by approximating the two incision borders, and the skin was approximated and fixed with methacrylate glue.

The heart was sectioned to expose its chamber lumen and immersed in 10% buffered formalin solution for inspection the next day. One of the lungs (the lung with better preservation of the hilar structures) was fixed with gaseous formaldehyde for 2 consecutive days at 30 cm H_2_O of transpulmonary pressure. The lung was then subjected to conventional CT (ex-situ CT). After macroscopic inspection and photographic documentation, representative samples were subjected to routine histological processing. The other lung was sampled in the autopsy room for molecular studies (samples stored at -80^o^C), and then fixed with an intratracheal injection of formalin for 24h for conventional histological procedures.

## RESULTS

**Figure 1:**
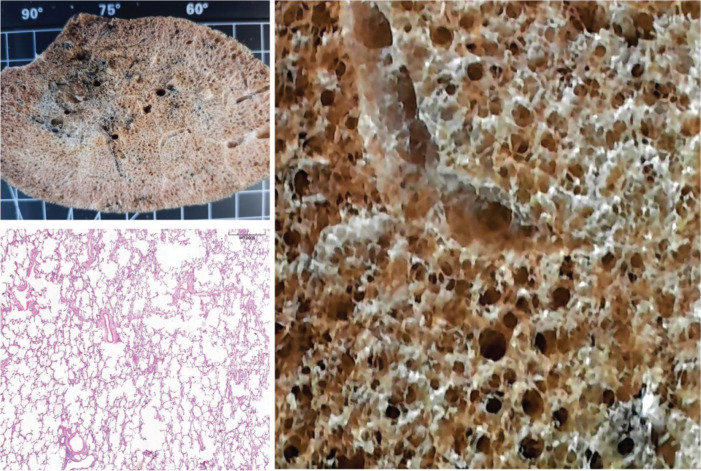
Example of a non-COVID-19, preserved lung, fixed with gaseous formaldehyde at 30 cm H_2_O for 48h. Lung parenchyma shows preserved architecture and normal histology, except for areas of anthracosis (black dots in upper left panel).

**Figure 2:**
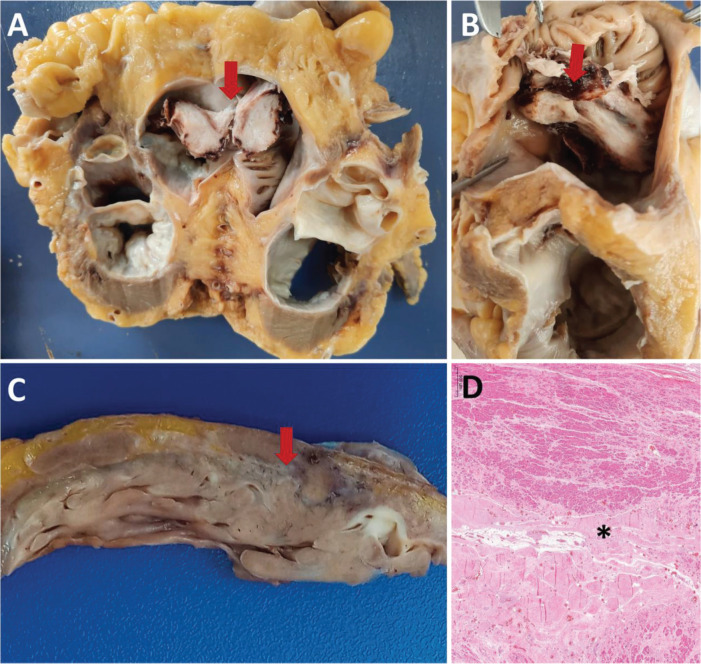
**A** and **B**: Male, 56 years old, 30 days of hospitalization. **A**: Cross section of the heart at the atrium-ventricle transition. Bottom view of an organized thrombus in the right atrium (arrow). **B**: Upper view of the organized thrombus in the right atrium (arrow). **C** and **D**: Male, 76 years old, 22 days of hospitalization. Gross section **(C)** and histology **(D)** of the left ventricle showing scarring areas of ischemic heart disease (arrow in **C** and asterisk in **D**).

Figure 1 shows a non-COVID-19 preserved lung, fixed with gaseous formaldehyde at 30 cm H_2_O for 48h. EMIA-US was performed in eight patients who died of severe COVID-19. Figures 2-6 show examples of cardiopulmonary events that could be evaluated using EMIA-US in these patients.

## DISCUSSION

This study presents a descriptive report of a new technical development for conducting non-conventional clinical autopsies. EMIA-US combines transcutaneous image-guided post-mortem biopsies with reduced thoracotomy to investigate cardiopulmonary involvement in the disease. EMIA-US was created owing to the need to investigate thrombotic and ischemic cardiopulmonary events, which are important aspects of the pathogenesis of severe COVID-19. The set of images presented in this report illustrates the potential of this procedure.

EMIA-US can be safely conducted in spaces without a conventional autopsy facility. Bleeding is minimal and can be efficiently controlled using a simple surgical aspirator to reduce the risk of contagion. Thus, EMIA-US fulfills one of the main advantages of MIA, that is, the ability to conduct comprehensive post-mortem investigation of human diseases in areas or sites devoid of autopsy facilities.

**Figure 4 f04:**
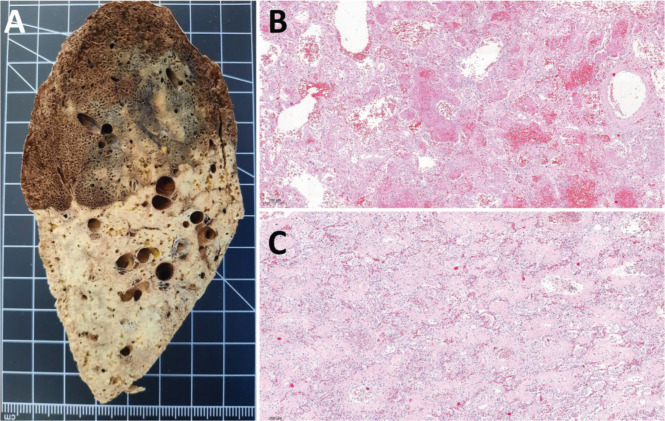
Female, 42 years old, 38 days of hospitalization and mechanical ventilation. **A**: Left inferior lung lobe shows marked transition between upper and lower regions, representing two different patterns of lung injury. Upper region corresponds to acute lung injury and lower region to advanced lung remodeling with fibrosis. **B**: Acute lung injury, with predominance of exudative changes, corresponds to the upper left inferior lobe shown in **A**. **C**: Remodeling of lung parenchyma with extensive fibrosis corresponds to the lower left inferior lobe shown in **A**.

**Figure 5 f05:**
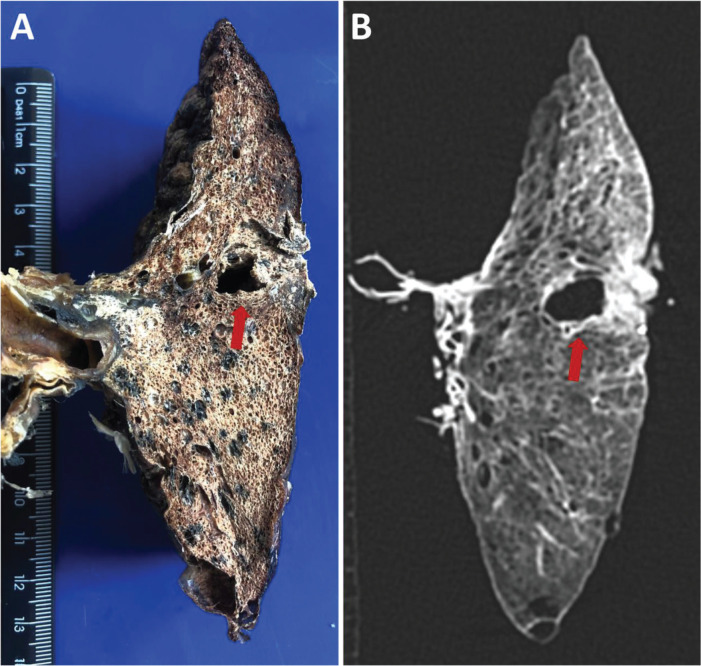
Male, 89 years old, 7 days of hospitalization, same patient as in Figure **3A** and **3B**. Ex-situ computed tomography **(B)** showing perfect correlation with the macroscopic evaluation of the lung **(A)**, ground-glass opacities, peripheral reticular pattern, and an area of lung cavitation (arrow in **A** and **B**), likely resulting from a previous abscess, with fistula formation toward the pleura.

**Figure 6:**
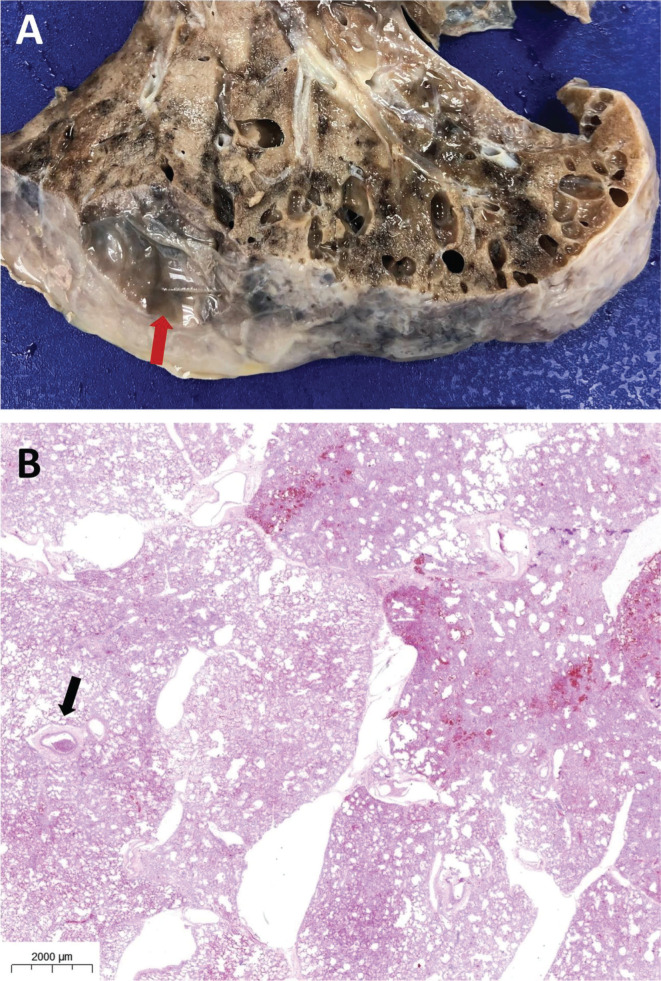
Female, 17 years old, 20 days of hospitalization, 16 days of mechanical ventilation. Bilateral pneumothorax and subcutaneous emphysema in the chest and face reported 9 days before death. **A**: Multiple areas of pulmonary interstitial emphysema secondary to barotrauma. Note a ruptured subpleural bubble (arrow). **B**: Pulmonary interstitial emphysema; air dissects along connective tissue sheaths of the bronchovascular bundles and interlobular septa. Arrow: arteriolar thrombus.

The possibility of larger tissue sampling than that in post-mortem biopsies performed using conventional MIA-US was useful to provide a clearer picture of the magnitude and severity of COVID-19, as exemplified in Figures 2-6. In addition to the original objective—a better evaluation of thromboembolic events and myocardial infarction—EMIA-US was useful for identifying the magnitude of pulmonary damage induced by mechanical ventilation (Figure 6) and the various aspects of COVID-19 progression (Figure 4). While conducting EMIA-US, we improved the technical skills of our team up to the point of being able to extract the entire lung with intact pleural coverage, creating the possibility of performing lung fixation with formaldehyde vapor. Having an air-filled and fixed lung, we made it possible to obtain, to the best of our knowledge, the first ex-situ lung CT images of a patient with COVID-19 (Figure 5). The possibility of combining ex-situ CT, macroscopic, and microscopic sets of images creates conditions to elaborate a pictorial atlas of lung diseases, designed to improve medical education.

## CONCLUSIONS

Minimally invasive image-guided post-mortem tissue sampling is a flexible and practical method to conduct post-mortem studies of human diseases, mainly in areas devoid of autopsy facilities or, alternatively, when autopsy is not possible owing to financial constraints, cultural and religious values, or for safety reasons, as in the case of highly contagious infectious diseases. Because of its practicality and mobility, it can be expanded to other areas of Brazil with very scarce resources (central and northern regions of the country), expanding the tools for sanitary surveillance of high-impact infectious diseases. We present evidence that an Extended MIA-US technique is feasible and can be used as an alternative to increase the accuracy of MIA-US in detecting cardiopulmonary events involving large vessels, which may not be assessed through post-mortem biopsies. Notably, MIA may revive the practice of clinical-pathological grounds in medical schools, which is, in our best judgment, essential for solid medical education.

## AUTHOR CONTRIBUTIONS

Theodoro-Filho J, Monteiro RAA and Saldiva PHN performed the autopsies. Duarte-Neto AN, Mauad T, Silva LFF, Saldiva PHN and Dolhnikoff M performed microscopic analysis and diagnostic interpretation. Saldiva PHN and Dolhnikoff M drafted the manuscript. All authors approved the final version of the manuscript. The authors participated sufficiently in the work to take public responsibility for appropriate portions of the content.

## Figures and Tables

**Figure 3 f03:**
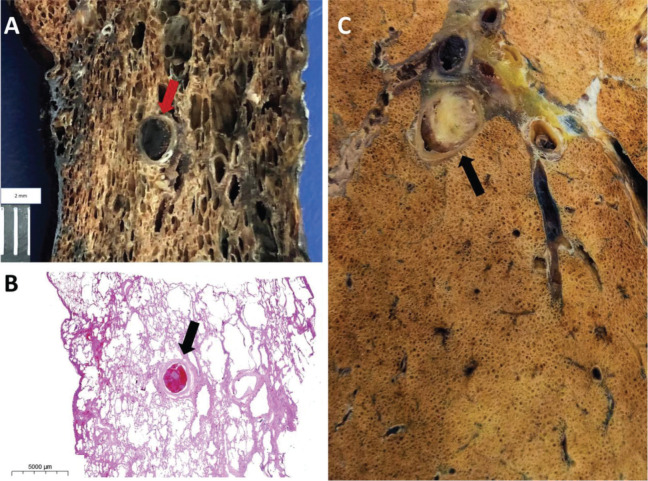
**A** and **B**: Male, 89 years old, 7 days of hospitalization. Gross section **(A)** and histology **(B)** of thromboembolism in a medium-sized (3.0 mm in diameter) pulmonary artery (arrow in **A** and **B**). **C**: Male, 76 years old, 24 days of hospitalization. Same patient as in Figure **2C and 2D**. Thromboembolism in a medium-sized (4.0 mm in diameter) pulmonary artery (arrow).

## References

[1] Cox JA, Lukande RL, Kalungi S, Van Marck E, Van de Vijver K, Kambugu A (2014). Needle autopsy to establish the cause of death in HIV-infected hospitalized adults in Uganda: a comparison to complete autopsy. J Acquir Immune Defic Syndr.

[2] Paganelli CR, Goco NJ, McClure EM, Banke KK, Blau DM, Breiman RF (2020). The evolution of minimally invasive tissue sampling in postmortem examination: a narrative review. Glob Health Action.

[3] Prata A (2000). Yellow fever. Mem Inst Oswaldo Cruz.

[4] Wong EB, Omar T, Setlhako GJ, Osih R, Feldman C, Murdoch DM (2012). Causes of death on antiretroviral therapy: a post-mortem study from South Africa. PLoS One.

[5] Duarte-Neto AN, Monteiro RAA, Johnsson J, Cunha MDP, Pour SZ, Saraiva AC (2019). Ultrasound-guided minimally invasive autopsy as a tool for rapid post-mortem diagnosis in the 2018 Sao Paulo yellow fever epidemic: Correlation with conventional autopsy. PLoS Negl Trop Dis.

[6] Duarte-Neto AN, Monteiro RAA, da Silva LFF, Malheiros DMAC, de Oliveira EP, Theodoro-Filho J (2020). Pulmonary and systemic involvement in COVID-19 patients assessed with ultrasound-guided minimally invasive autopsy. Histopathology.

[7] Dolhnikoff M, Duarte-Neto AN, de Almeida Monteiro RA, da Silva LFF, de Oliveira EP, Saldiva PHN (2020). Pathological evidence of pulmonary thrombotic phenomena in severe COVID-19. J Thromb Haemost.

[8] Mauad T, Duarte-Neto AN, da Silva LFF, de Oliveira EP, de Brito JM, do Nascimento ECT (2021). Tracking the time course of pathological patterns of lung injury in severe COVID-19. Respir Res.

[9] Duarte-Neto AN, Caldini EG, Gomes-Gouvêa MS, Kanamura CT, de Almeida Monteiro RA, Ferranti JF (2021). An autopsy study of the spectrum of severe COVID-19 in children: From SARS to different phenotypes of MIS-C. EClinicalMedicine.

[10] Monteiro RAA, Duarte-Neto AN, Silva LFFD, Oliveira EP, Filho JT, Santos GABD (2020). Ultrasound-guided minimally invasive autopsies: A protocol for the study of pulmonary and systemic involvement of COVID-19. Clinics (Sao Paulo).

